# Surface antigens of *Plasmodium falciparum*-infected erythrocytes as immune targets and malaria vaccine candidates

**DOI:** 10.1007/s00018-014-1614-3

**Published:** 2014-04-02

**Authors:** Jo-Anne Chan, Freya J. I. Fowkes, James G. Beeson

**Affiliations:** Burnet Institute, Melbourne, VIC Australia

**Keywords:** *Plasmodium falciparum*, Variant surface antigens, Antibodies, PfEMP1, Malaria, Vaccines

## Abstract

Understanding the targets and mechanisms of human immunity to malaria caused by *Plasmodium falciparum* is crucial for advancing effective vaccines and developing tools for measuring immunity and exposure in populations. Acquired immunity to malaria predominantly targets the blood stage of infection when merozoites of *Plasmodium* spp. infect erythrocytes and replicate within them. During the intra-erythrocytic development of *P. falciparum*, numerous parasite-derived antigens are expressed on the surface of infected erythrocytes (IEs). These antigens enable *P. falciparum*-IEs to adhere in the vasculature and accumulate in multiple organs, which is a key process in the pathogenesis of disease. IE surface antigens, often referred to as variant surface antigens, are important targets of acquired protective immunity and include PfEMP1, RIFIN, STEVOR and SURFIN. These antigens are highly polymorphic and encoded by multigene families, which generate substantial antigenic diversity to mediate immune evasion. The most important immune target appears to be PfEMP1, which is a major ligand for vascular adhesion and sequestration of IEs. Studies are beginning to identify specific variants of PfEMP1 linked to disease pathogenesis that may be suitable for vaccine development, but overcoming antigenic diversity in PfEMP1 remains a major challenge. Much less is known about other surface antigens, or antigens on the surface of gametocyte-IEs, the effector mechanisms that mediate immunity, and how immunity is acquired and maintained over time; these are important topics for future research.

**Electronic supplementary material:**

The online version of this article (doi:10.1007/s00018-014-1614-3) contains supplementary material, which is available to authorized users.

## Introduction

**Fig. 1:**
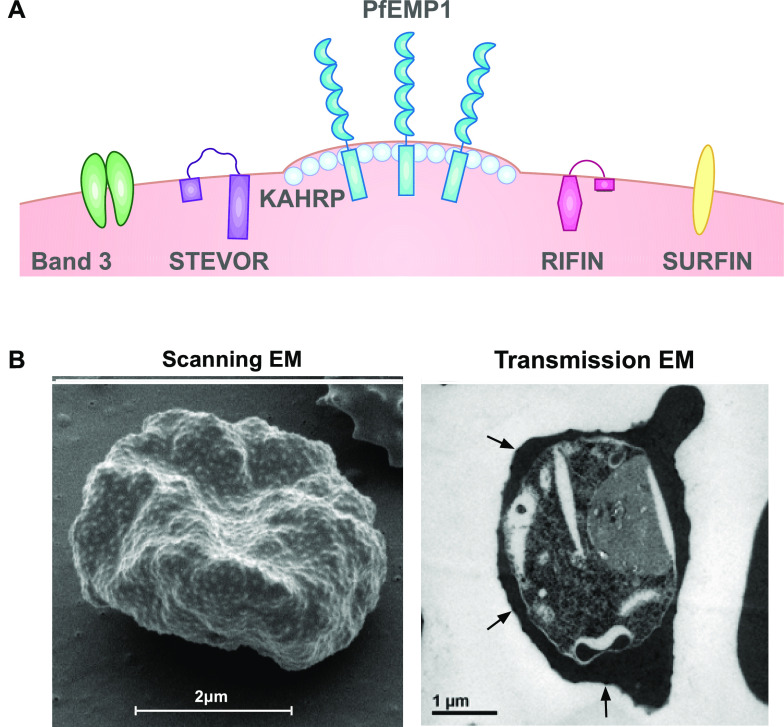
Parasite-induced modifications to *P. falciparum*-infected erythrocytes. A. During intra-erythrocytic development, *P. falciparum* expresses knob structures and VSAs on the surface of pigmented trophozoite IEs. PfEMP1, *P. falciparum* erythrocyte membrane protein 1; RIFIN, repetitive interspersed family; STEVOR, subtelomeric variable open reading frame; SURFIN, surface-associated interspersed gene family; KAHRP, knob-associated histidine-rich protein. B. Scanning (*left*) and transmission (*right*) electron microscopy (EM) shows the ultrastructural features of the IE membrane. The IE membrane is distorted by surface knob protrusions (*arrows*) that present the major virulence factor, PfEMP1

*Plasmodium falciparum* is the most virulent form of human malaria and is a leading cause of mortality among children under 5 years [1]. *Plasmodium falciparum* has a complex lifecycle involving a mosquito vector and a human host. The on-going asexual reproduction during the blood stage leads to clinical symptoms of malaria [2]. The pathogenesis of human malaria stems from various host and parasite factors that concurrently influence the severity and outcome of disease. Key pathophysiological features include the sequestration of *P. falciparum*-infected erythrocytes (IEs) in the microvasculature, the induction of proinflammatory cytokines and anemia resulting from the suppression of erythropoiesis [2, 3]. The destruction of uninfected erythrocytes and IEs further compromises oxygen delivery and exacerbates disease pathogenesis [4]. An important virulence property of *P. falciparum* is the expression of parasite-derived antigens on the surface of IEs, generally known as variant surface antigens (VSAs; Fig. 1), and its strong propensity to adhere in the vasculature. VSAs are comprised of novel parasite-derived proteins and include *P. falciparum* erythrocyte membrane protein 1 (PfEMP1) [5], repetitive interspersed family (RIFIN) proteins [6–8], sub-telomeric variable open reading frame (STEVOR) proteins [9–11], surface-associated interspersed gene family (SURFIN) proteins [12] and possibly others such as *P. falciparum* Maurer’s cleft two transmembrane (PfMC-2TM) proteins [13, 14]. Parasite-modified erythrocyte band 3 has also been proposed as a surface antigen or ligand for IE sequestration [15, 16]. These IE surface proteins are antigenically diverse and undergo clonal antigenic variation because of the selective pressure exerted by human immunity. The significance of VSAs as targets of naturally acquired immunity and their potential as vaccine candidates is the focus of this review. Acquired immunity to blood stage *P. falciparum* will be addressed, followed by a summary of the VSAs expressed on the IE surface and finally human antibodies to different VSA families.

### *Plasmodium falciparum* sequestration and cytoadhesion

The virulence of *P. falciparum* malaria is attributed to the adhesion of IEs to the vascular endothelium or to uninfected erythrocytes to form rosettes [17–19]. Mature *P. falciparum* disappear from the peripheral circulation and are sequestered in various organs throughout the body. The importance of splenic clearance of IEs in controlling disease severity has been demonstrated by numerous studies. For example, a study conducted with *Aotus* monkeys showed that splenectomised animals developed virulent infections, presumably because of enhanced accumulation of IEs in the microvasculature [20, 21]. IE sequestration contributes to the pathogenesis of severe disease syndromes such as cerebral [17, 22, 23] and placental complications [24, 25]. An important feature of IEs that enables *P. falciparum* to sequester is the expression of knob structures on the IE membrane [26–28]. The major structural component of knobs is the knob-associated histidine-rich protein (KAHRP) [27, 29–32]. Other parasite-encoded proteins such as *P. falciparum* erythrocyte membrane protein 3 (PfEMP3) [33] and mature IE surface antigen (MESA; also known as PfEMP2) [34, 35] also contribute to knob assembly. KAHRP interacts with cytoskeletal components of the erythrocyte such as spectrin and actin [36–38], resulting in reduced membrane deformability [39]. Knobs present the major virulence factor, PfEMP1 [5], on the external surface of the IE membrane, where it mediates IE cytoadhesion to the host endothelium under physiological flow conditions [40, 41]. Disruption of the *kahrp* gene impairs proper knob formation and leads to a decrease in surface-exposed PfEMP1 and reduced cytoadhesion [42]. However, the presence of knobs may not necessarily result in sequestration [43]; *P. malariae* has knob structures but does not sequester, while *P. chabaudi* sequesters without knobs [16, 43].

A diverse range of host receptors that mediate IE cytoadhesion has been identified [44–46]. The main parasite ligand responsible for cytoadhesion is PfEMP1 and it binds to a range of endothelial and erythrocyte molecules including CD36 [47], ICAM-1 [48], chondroitin sulphate A (CSA) [49, 50], complement receptor 1 (CR1) [51], heparan sulfate (HS) [52] and others. IEs are capable of binding via multiple receptors [53] therefore creating a synergistic effect on IE adhesion [54]. Most *P. falciparum* isolates adhere to both ICAM-1 and CD36, which are widely distributed in the vasculature [53, 55, 56], but parasites isolated from infected placentas mainly adhere to specific receptors expressed by the syncytiotrophoblasts of the infected placenta [57, 58], particularly CSA [56, 59, 60], and possibly secondary receptors such as hyaluronic acid (HA) [61–63] and non-immune IgM [64–66] and IgG [67]. The differential expression of endothelial cell receptors in various tissues leads to the preferential binding of IEs. For example, it is proposed that ICAM-1-binding parasites are more likely to sequester in the brain [46, 68] as the brain endothelium expresses ICAM-1. While it has been speculated that receptor-specific adhesion (e.g. ICAM-1) predisposes to a particular pattern of disease (e.g. cerebral malaria), studies to date have been inconclusive. In one study, cerebral malaria patients did not show a significant association between disease and ICAM-1 binding [69], and another study reported that ICAM-1-binding was lowest in children with severe malaria [70]. In contrast, post-mortem histopathological analyses of infected cerebral vessels proposed a role for ICAM-1 in the manifestation of severe disease [68], and another study demonstrated that ICAM-1-binding was greater in cerebral malaria patients compared to patients with uncomplicated malaria [71]. This may propose a role for ICAM-1 in the pathogenesis of cerebral malaria but additional studies are necessary to further validate this association. A recent study identified endothelial protein receptor C as a likely mediator of cerebral sequestration [72]. Other host receptors implicated in IE cytoadherence include thrombospondin (TSP) [73], platelet/endothelial cell adhesion molecule (PECAM/CD31) [74], P-selectin [75] and vascular cell adhesion molecule-1 (VCAM-1) [53], but the significance of these receptors in disease pathogenesis remains unclear.

The clustering of mature IEs to uninfected erythrocytes, known as rosetting [76], is also thought to contribute to excessive microvasculature obstruction [77, 78]. Rosetting is associated with severe malaria in African children, suggesting that it contributes to disease pathogenesis [79–83]. However, a study with Malawian [70] and PNG [84] children reported that the rosetting occurred at a similar rate between children with severe and uncomplicated malaria, suggesting that rosette formation is not always associated with severe clinical outcomes. The parasite ligand for rosetting in *P. falciparum* has been identified as a specific PfEMP1 variant that binds to CR1 [51] or HS proteoglycans [85] expressed by the host erythrocyte.

*Plasmodium falciparum* also causes vascular obstruction through the clumping of IEs, a feature that was first reported as autoagglutination [86]. This adhesive phenotype is distinct from rosetting, as autoagglutinating parasites do not form rosettes and rosetting parasites do not autoagglutinate [86]. Autoagglutination was a common feature of infection, although more autoagglutinates were observed in children with severe malaria compared to those with mild malaria. This suggests that autoagglutination is more frequently observed in, but not restricted to, severe disease [87]. It was later reported that autoagglutination is mediated by platelets and required the expression of the platelet glycoprotein CD36 [88]. Scanning electron microscopy of platelet-mediated clumping of IEs showed that this interaction occurred at the IE knob structures [88]. Studies with Kenyan children [88] and patients from Thailand [89] established that platelet-mediated clumping of IEs was associated with severe disease, presumably through local disruptions of blood flow. Conversely, binding of platelets to IEs has also been implicated in protection against *P. falciparum* by directly inhibiting intra-erythrocytic parasite growth [90, 91].

## VSAs of *P. falciparum*

The most extensively studied VSA is the major virulence factor PfEMP1, an important target of naturally acquired immunity [92, 93]. The *var* genes that encode PfEMP1 appear to be unique to *P. falciparum*, but the *P. knowlesi* schizont-infected cell agglutination (SICA) antigens encoded by the *SICAvar* multigene family [94, 95] have been described as conceptually similar to PfEMP1 [96]. Orthologs of *rif* and *stevor* genes have been identified in other *Plasmodium* species, known collectively as the *pir* multigene family (*Plasmodium* interspersed repeats). These include the *vir* multigene family in *P. vivax* [97], *kir* multigene family in *P. knowlesi*, *yir* multigene family in *P. yoelii*, *bir* multigene family in *P. berghei* and *cir* multigene family in *P. chabaudi* [98, 99].

Currently, little is known regarding the mechanisms that regulate gene transcription in *P. falciparum* other than the involvement of specific transcription factors and promoter interactions. Exploiting the mutually exclusive transcription of *var* genes has allowed for the suppression of the entire endogenous *var* multigene family [100, 101], thus enabling the specific study of PfEMP1 [102]. Conversely, disrupting up to 150 genes such as in the *rif* family is not currently feasible, and knockdowns of *rif*, *stevor* and *pfmc2tm* gene families have not been achieved. These multigene families share a common activation factor necessary for gene expression, and it has been proposed that the downregulation of one multigene family may affect the expression of members of other multigene families in some conditions [103]. A transcriptionally active *rif* promoter co-localised with an active *var* promoter and the downregulation of members in the *stevor* multigene family appeared to increase transcription of the *pfmc*-*2tm* multigene family [103]. Further studies must be employed to dissect the functional roles of these multigene families.

It is hypothesised that a large proportion of parasite proteins exported into the host erythrocyte supports the correct trafficking and surface display of PfEMP1 and other erythrocyte surface proteins. This also includes alterations in the spectrin network and knob protrusions at IE membrane [104]. Many exported proteins contain a pentameric sequence, known as the *Plasmodium* export element (PEXEL) [105] or vacuolar translocation signal [106], required for the translocation of proteins across the PV membrane. Exported parasite proteins such as PfEMP1 are trafficked via the translocon complex (PTEX; *Plasmodium* translocon of exported proteins) located at the parasitophorous vacuole (PV) [107, reviewed in 108]. Most parasite proteins are destined for the IE cytosol, and only a small portion is exposed on the IE surface. Interestingly, recent studies have showed that exported parasite proteins may play a role in cellular communication between IEs through microvesicles [109] and exosome-like vesicles [110]. Microvesicles lack components of the knob structure like KAHRP and PfEMP1, suggesting that they originate from MC structures or regions of the erythrocyte membrane that exclude knobs [109]. It was further demonstrated that PfEMP1 is not required for efficient intercellular communication as modified parasites with inhibited PfEMP1 expression were still able to receive exosome-like vesicles [110].

### PfEMP1 and *var* genes

PfEMP1 was first identified by immunoprecipitation with immune sera from infected *Aotus* monkeys [5] and is encoded by the highly polymorphic *var* multigene family (~60 genes per genome) [40, 111, 112]. Through mutually exclusive transcription of *var* genes, only a single PfEMP1 variant is generally expressed on the IE surface at a given time [52, 113]. However, a recent study reported the potential expression of more than one PfEMP1 variant on the IE surface as demonstrated by live confocal microscopy, in vitro adhesion assays and cell sorting by flow cytometry [114]. PfEMP1 is a high-molecular-weight protein (200–350 kDa) and is highly sensitive to cleavage by mild trypsin treatment of intact IEs (10 μg/ml) [5]. The biochemical properties of PfEMP1 (Triton X-100-insoluble and SDS-soluble) demonstrate its anchorage to the IE membrane [115].

The export of PfEMP1 is a highly complex process due to its large size, number of membranes to traverse before reaching the IE surface and the involvement of various chaperone proteins (reviewed in [116, 117]). PfEMP1 molecules are associated with the Maurer’s clefts (MCs) and are ultimately presented by the knob structures at the IE surface at approximately 18 h post-invasion [5, 32, 115, 118–120]. The mechanism of transport of PfEMP1 from the parasite to the IE surface as well as its partner proteins involved remains a process that is poorly defined.

An important MC-resident protein essential for PfEMP1 trafficking is the 48 kDa *P. falciparum* skeleton binding protein 1 (SBP1) [121–123]. Disruption of the *pfsbp1* gene impaired the loading of PfEMP1 molecules into the MCs and resulted in the loss of surface-exposed PfEMP1, but the trafficking of other MC proteins such as KAHRP, MAHRP1 and REX1 was unaffected in the transgenic parasites [122, 123]. A large-scale gene knockout screen further identified MC proteins termed *P. falciparum* PfEMP1 trafficking protein (PfPTP) involved in the trafficking of PfEMP1 to the IE surface [124]. Of the 83 *P. falciparum* genes that were disrupted, 6 genes were specifically found to affect the export and surface display of PfEMP1, and 2 were found to disrupt proper knob formation. In these transgenic parasites, PfEMP1 export was arrested either at the PVM or the MC structures in the IE cytosol [124]. Among the proteins involved in the trafficking of PfEMP1 are members of the HSP40/DNAJ and PHIST family [124]. Recently, an exported parasite-encoded HSP70 known as PfHSP70-x was found to complex with HSP40s and colocalised with PfEMP1 in the IE cytosol [125]. Other MC-associated proteins that have been proposed to play a role in the trafficking of PfEMP1 include MAHRP [126], REX1 [127] and Pf332 [128].

Transcription of *var* genes is epigenetically regulated by the SIR complex as gene disruption of *PfSIR2* results in activation of multiple members of this multigene family [129–131]. The rapid switching rate of *var* genes, of up to 2 % per generation [86], was demonstrated to correlate with changes in IE adhesive and antigenic phenotypes [112, 132]. The typical structure of PfEMP1 includes the variable N-terminal segment (NTS) exposed on the IE surface to interact with host receptors [133], a transmembrane domain and a conserved acidic terminal segment (ATS) [48]. The cytoplasmic ATS domain interacts with KAHRP [134–136], thus anchoring PfEMP1 to the IE membrane. The extracellular portion of PfEMP1 consists of Duffy binding-like (DBL) adhesive domains, the C2 domain and the cysteine-rich interdomain regions (CIDR) [48]. DBL domains are grouped as five sequence classes (α, β, γ, δ, ε), and CIDR domains are grouped as three distinct classes (α, β, γ). While the number of CIDR and DBL domains may vary between different PfEMP1 variants, certain domain architectures such as DBLαCIDRα or DBLδCIDRβ are preferred. This conservation may reflect the biological function of PfEMP1 [48]. The binding site of ICAM-1 resides within the DBL2β and c2 regions of PfEMP1 [137, 138], while the binding site of CD36 is mapped to the CIDRα region [47, 139, 140]. The binding site of CSA in pregnancy-associated parasites lies within the DBL1-DBL3 domain of PfEMP1 [141].

The *var* genes can be classified into three main subgroups based on their upstream promoter regions (upsA, upsB and upsC) and the single-copy conserved intergenomic genes *var1* and *var2csa* [142–145]. The *var* gene repertoires of clinical isolates can also be classified according to short sequence tags amplified from the DBLα domain [146, 147]. Analyses of the DBLα sequences from Kenya showed the presence of two or four cysteine residues, with a minority containing one, three, five or six cysteines; therefore, *var* genes were sub-grouped according to cys2, cys4 and cysX, respectively [147]. These DBLα sequences were further classified according to the amino acid motifs occurring at four fixed positions within the sequenced region, known as “positions of limited variations” (PoLV1-4) [147]. Expression of *var* genes from the cys2 group was associated with severe malaria in young children [148, 149]. The differential transcription of *var* gene subgroups has been linked to clinical disease. Transcription of group A *var* genes was also associated with rosetting parasites [150] and severe malaria in African children [151–153]. Furthermore, the elevated expression of group A-like *var* genes was associated with impaired consciousness, a key feature of severe disease [154]. Recent studies also reported the expression of a restricted subset of *var* genes encoding PfEMP1 variants that bind human brain endothelial cells [155, 156]. These *var* genes belong to group B/A genes that are expressed in early childhood infections [148, 157] and are associated with more severe infections [149, 151]. PfEMP1 variants from group B and C *var* genes are also associated with autoagglutination and ICAM-1-binding, features that contribute to severe disease [133, 138]. The lack of association between transcription of *var* group C and clinical presentation suggests that perhaps these *var* genes are involved in the establishment of chronic infections [158]. These findings support the correlation between *var* gene expression patterns and clinical presentations, thus suggesting that protective immunity could be conferred by antibodies to key *var* gene subgroups [147, 158].

A specific *var* gene, *var2csa,* is relatively conserved in sequence and is present as a single-copy gene in most isolates. However, some isolates have more than one copy of *var2csa* (e.g. HB3 has two copies of *var2csa*) [159–161]. This gene is upregulated in placental isolates and mediates IE adhesion to CSA and other placental receptors such as HA and immunoglobulins [162–165]. Polyclonal antibodies generated against recombinant domains of VAR2CSA recognised the IE surface of parasites isolated from infected placental tissue [166]. Furthermore, sera from pregnant women recognised the IE surface of VAR2CSA-expressing parasites in a parity-dependent manner [164, 167]. Pregnant women with elevated levels of these antibodies had a reduced risk of delivering low-birth-weight babies [164]. Targeted gene disruption of *var2csa* in the isolates FCR3 [168] and 3D7 [169] inhibited CSA adhesion, suggesting the central role of *var2csa* in mediating placental adhesion. In contrast, disruption of *var2csa* in CS2 parasites also ablated CSA binding but repeated selection on CSA restored their binding ability [169] presumably through the expression of other PfEMP1 variants proposed to bind CSA [49]. The use of different parasite lines may reflect the discrepancies between these studies and that the FCR3 or 3D7 isolates lack the PfEMP1 variants thought to rescue the CSA-binding ability.

The *var* transcripts have also been detected by RT-PCR in both immature (I–II) and mature stage gametocyte-IEs (IIB–V). Initial studies suggested that the *var* genes transcribed in gametocyte-IEs were similar or identical to those expressed by asexual parasites [170]. However, it was later discovered that the *var* transcript profile was unlinked to their asexual progenitors. Furthermore, it appears that the most abundant *var* transcripts found in gametocyte-IEs (generated in vitro) belong to the non-subtelomeric group C *var* genes [171]. Data regarding the pattern of PfEMP1 expression in gametocyte-IEs are conflicting. Early studies reported the expression of PfEMP1 in all five stages of gametocyte development but with stage-specific patterns. PfEMP1 staining was visualised at the IE membrane of immature gametocyte-IEs (stages I–IIA) but not of mature gametocyte-IEs (IIB–V) [170]. A recent study reported low levels of PfEMP1 expression on the surface of immature gametocyte-IEs, which was absent in mature gametocyte-IEs [172]. Thus, it is thought that sequestration of immature gametocyte-IEs is mediated by PfEMP1, after which its role is replaced by an alternative ligand present on the surface of mature gametocyte-IEs or through mechanical effects [173].

### RIFIN proteins

The *rif* multigene family (150–200 genes per genome) encoding a group of clonally variant RIFIN proteins represents the largest multigene family identified in *P. falciparum* [6–8]. Transcription of *rif* genes occurs approximately 12 h post-invasion, but RIFIN proteins are thought to appear on the IE surface at the same time as PfEMP1 [174]. In contrast to *var* genes, a single parasite simultaneously transcribes several *rif* genes, resulting in the expression of multiple RIFIN variants on the IE surface [8]. All RIFIN sequences contain the PEXEL motif required for correct export [105]. Surface exposure of RIFIN was evident from immunoprecipitation and Western blot analyses. Bands of expected size corresponding to RIFIN (30–45 kDa) were absent after IEs were treated with high concentrations of trypsin (>100 μg/ml), a concentration much greater than that needed to cleave the highly trypsin-sensitive PfEMP1 [7, 8]. Some variants of the large RIFIN family are also expressed in other developmental stages such as merozoites, sporozoites and gametocytes [175–177]. Bioinformatic analyses of RIFIN sequences revealed two major subgroups of the RIFIN family [178] that are simultaneously expressed in a single parasite. A-type RIFINs associate with the MCs and are destined for the IE surface, whereas B-type RIFINs remain confined within the parasite [176]. Although the biological function of RIFIN remains unknown, the exposure of their highly polymorphic V2 epitope on the IE surface suggests they contribute to antigenic variation of *P. falciparum* [6, 179]. Although direct evidence is lacking, RIFIN was proposed to play a role in rosetting [7, 8].

### STEVOR proteins

The *stevor* multigene family, encoding STEVOR proteins (~30–40 kDa), is the third largest identified in *P. falciparum* (reviewed in [10]). First described as 7h8, *stevor* was detected by a monoclonal antibody as an expressed sequence [6]. Each parasite genome is predicted to contain approximately 30–40 copies of *stevor* genes. Like *var* and *rif*, *stevor* genes are located at the telomeres of most *P. falciparum* chromosomes [6]. Similar to *rif*, multiple *stevor* transcripts were detected in a single parasite. Peak *stevor* transcription occurs at 28 h post-invasion during late trophozoites and early schizonts, where they appear to localise in the IE cytosol. As the parasite matures, STEVOR co-localises with PfSBP1 and PfEMP3 at the MCs [9, 180] in immunofluorescence microscopy with fixed IEs [10, 11]. Immunofluorescence microscopy with live, intact schizont stage IEs suggests the surface localisation of STEVOR, which was removed upon IE trypsinisation [11, 181]. A recent study demonstrated that *stevor* overexpression contributes to increased IE rigidity [182], together with other IE cytoskeletal members such as RESA [183]. It is proposed that the STEVOR-increased stiffness of IEs enhances PfEMP1-mediated IE sequestration [182]. The biological function of STEVOR remains unclear. Because STEVOR is clonally variant [13, 184], it may be involved in immune evasion concurrently with PfEMP1 and RIFIN [10, 11, 13]. In addition, STEVOR has been proposed to play a role in parasite invasion [185]. STEVOR proteins are also expressed in merozoites [10, 186, 187], sporozoites [175] and gametocytes [9]. Interestingly, the same STEVOR variants are transcribed in gametocytes and their asexual progenitors, suggesting that perhaps STEVOR plays a similar role in these lifecycle stages [171].

### SURFIN proteins

Little is known about the *surf* multigene family (10 genes), which encodes high-molecular-weight antigens (~280–300 kDa) known as SURFIN proteins [12]. The expression of *surf* genes is differentially transcribed according to different stages of the intra-erythrocytic parasite. The expression of *surf*_1.3_, *surf*_4.2_ and *surf*_8.3_ genes was detected throughout parasite development while other *surf* genes were either not detected or restricted to later developmental stages [188]. A variant expressed by 3D7 and FCR3 parasites, SURFIN_4.2_ was identified by mass spectrometric analysis of proteins cleaved off the surface of intact IEs by trypsin [12]. SURFIN_4.2_ was only detected in a subpopulation of cultured IEs (~25 %) with increasing protein expression during mature developmental stages (24–44 h post-invasion). Immunoelectron microscopy showed the presence of SURFIN_4.2_ at the knob structures suggesting its co-localisation with PfEMP1 at the IE surface [12]. However, attempts to verify the surface localisation of SURFIN_4.2_ proteins with live, intact IEs were inconclusive. Another variant, SURFIN_4.1_, localised to the parasitophorous vacuole (PV) but not within the erythrocyte cytosol in mature IEs (>30 h post-invasion) [188]. SURFIN antibodies did not agglutinate mature IEs and no fluorescence was observed with live IEs, suggesting that SURFIN_4.1_ is not exposed on the IE surface [188]. Whether SURFIN proteins potentially elicit humoral immunity or mediate immune evasion has not been determined.

### Other membrane proteins

*Plasmodium falciparum* Maurer’s clefts two-transmembrane protein (PfMC-2TM) is encoded by a novel gene family (~13 members) located at the subtelomeric regions of several *P. falciparum* chromosomes [14, 142]. PfMC-2TM is highly conserved within the N-terminus, both transmembrane domains and the C-terminus. The short loop between the transmembrane domains is highly polymorphic, similar to that proposed for RIFIN and STEVOR [13, 184]. The diversity within this loop region proposed the inclusion of *pfmc*-*2tm* as a variant multigene family together with *var*, *rif* and *stevor* [13, 14]. It has not been determined whether PfMC-2TM is associated with the IE membrane or exposed on the IE surface.

Another IE membrane protein is modified erythrocyte band 3, which has been proposed as a ligand for IE adhesion to CD36 [189] and thrombospondin [190, 191]. Chemical modifications of band 3 led to a reduction in CD36 binding but not thrombospondin, thus supporting its role in CD36 adhesion [189]. Synthetic peptides based on the exofacial loops of band 3 and antibodies generated against these peptides were capable of inhibiting IE adhesion to C32 amelanotic melanoma cells. Additionally, intravenous infusion of these peptides into *Aotus* and *Saimiri* monkeys infected with *P. falciparum* isolates prevented IE sequestration [192]. However, its significance in relation to PfEMP1 as an adhesive ligand remains unclear. In our recent study, parasites with suppressed PfEMP1 expression were found to retain a substantial proportion of CD36 binding, but not ICAM-1, thus raising the possibility that additional surface antigens contribute to CD36 adhesion [102].

## Naturally acquired immunity to malaria

Protective immunity to malaria is elicited through complex interactions between both humoral and cell-mediated responses [193–195]. This protection against symptomatic malaria in humans develops gradually after repeated exposure to *P. falciparum* infections (reviewed in [196]). In malaria-endemic areas, the risk of severe disease is greatest during the first few years of life, after which the risk rapidly declines as children begin to acquire natural immunity. A study of young African children reported that immunity to non-cerebral severe malaria may develop after several infections and is almost complete by the age of 5 [197]. Adolescents and adults eventually develop protection from severe illness and death, although sterile immunity is rarely or perhaps never achieved [198]. Maternal antibodies transferred across the placenta are also thought to confer protection in young infants [199, 200]. It is becoming increasingly clear that effective immunity to malaria involves immune responses to multiple antigens expressed at different parasite stages and requires multiple immune effector mechanisms [195]. It is likely that the development of a highly effective malaria vaccine will require the inclusion of multiple antigens and that single-antigen vaccines will not be optimally efficacious.

### Cell-mediated immunity to VSAs

While the significance of humoral immunity to *P. falciparum* is well established in humans, the role of cell-mediated immunity (CMI) remains poorly understood. CMI acts through complex interactions with the innate and adaptive immune response (reviewed in [195, 201]). Most studies of CMI have been based on murine models of malaria (reviewed in [202]). Early studies showed that mice incapable of making B cells have the ability to control infection [203], suggesting the importance of CMI in protection against malaria. Antigen-presenting cells process parasite antigens for display on major histocompatibility complex molecules to recruit antigen-specific CD4+ T cells. Th1 cells produce proinflammatory cytokines such as TNF-α and IFN-γ, which lead to monocyte activation and the release of toxic mediators that limit *P. falciparum* growth [204]. In a study of Gabonese children, IFN-γ responses to erythrocytic antigens were associated with lower rates of *P. falciparum* reinfection [205]. Despite this protective potential, CMI responses have also been implicated in disease pathogenesis and the development of severe malaria (reviewed in [206]).

Although data on CMI responses to VSAs are limited, studies have reported that parasites may modulate CMI to evade host immune responses. For example, the maturation of dendritic cells cultured in vitro was suppressed following exposure to erythrocytes infected with *P. falciparum* [207]. It was later reported that the modulation of dendritic cells was not dependent on the interaction between PfEMP1 and CD36 [208]. Interaction between IEs and natural killer (NK) cells leads to their activation, including production of IFN-γ [209]. A recent study using a humanised mouse model has reported that NK cell binding of IEs leads to the activation of NK cells and the elimination of IEs [210]. Using parasites with modified PfEMP1 expression, others reported that PfEMP1 appeared to suppress innate IFN-γ production by naïve CD4+ T cells and NK cells [211]. The CIDR1α domain of PfEMP1 was found to induce polyclonal B cell activation that contributes to the evasion of host immune responses [212–214] and stimulates CD4+ T cells from both malaria-exposed and non-exposed individuals [215]. The addition of recombinant CIDR1α to naïve human peripheral blood mononuclear cells resulted in the activation of CD4+ T cells and NK cells, leading to IFN-γ production [216]. It appears that a fine balance between protective immunity and immunopathology must be achieved in CMI. The lack of CMI-related studies in human malaria and the difficulty of inferring results from murine models are continuing obstacles in our understanding of the role of CMI and a priority topic for further research.

## Human antibodies to VSAs

The development of protective immunity to *P. falciparum* is characterised by a decrease in disease severity over several years after repeated infections [217]. Sterile immunity to *P. falciparum* is rarely achieved, as adults living in malaria-endemic regions remain susceptible to asymptomatic infection and often experience persistent low levels of parasitaemia without clinical disease [196, 198]. The passive transfer of gamma-globulin from immune individuals to *P. falciparum*-infected individuals confers protection against malaria infection [218]. Antibodies to both merozoite antigens [219–221] and VSAs appear to play an important role in mediating acquired immunity. The focus of this review is on VSAs, and the significance of merozoite antigens as immune targets is reviewed in detail elsewhere [222]. In brief, numerous antigens on the surface of merozoites (e.g. merozoite surface protein 1, 2, and 3) and erythrocyte invasion ligands (e.g. erythrocyte-binding antigens, PfRh invasion ligands and apical membrane antigen 1) have been identified as important targets of acquired immunity and promising vaccine candidates [219–221, 223, 224]. Although it is highly likely that antibodies to VSA and merozoite antigens contribute to immunity and a strong response to both types of antigens may be essential for highly effective immunity, there are few reports on the relationship between these responses and how they may interact to mediate immunity [225]; this is an important question for further research.

Naturally acquired antibodies against VSAs typically demonstrate a high degree of strain specificity [132, 226]. Antigenic diversity by *P. falciparum* enables repeated infections to occur over time, as new infections appear to exploit gaps in the repertoire of previously acquired variant-specific antibodies [226]. Antibodies to polymorphic VSAs expressed on the IE surface, such as PfEMP1, have been proposed to play a key role in mediating protective immunity [93, 226–228]. Most published studies used agglutination assays to describe antigenic variation in PfEMP1 as switches in the agglutination phenotype are correlated with switches in *var* gene expression [112] or PfEMP1 [132]. However, it is difficult to determine PfEMP1-specific antibody responses because of the number of antigens expressed on the IE surface; therefore, antibodies measured to the IE surface are hereafter classified as antibodies to all VSAs (studies summarised in Table S1).

Early studies that measured agglutination antibody responses to *P. falciparum* infections in Pakistan [229], Papua New Guinea [230] and Africa [226, 231] reported that children developed isolate-specific antibodies to VSAs after infection. Mixed agglutination assays that allowed the determination of shared epitopes on the IE surface also showed that acquired human antibodies are predominantly variant specific while cross-reactive antibodies are rare [231]. However, antibodies from convalescent sera from adults were capable of agglutinating diverse *P. falciparum* isolates [232], and antibodies acquired towards placental-binding parasites expressing VAR2CSA have a significant amount of cross-reactivity against different isolates, despite polymorphisms in VAR2CSA [159, 233]. Furthermore, an acute *P. falciparum* infection in returned travellers was sufficient to induce broadly cross-reactive antibodies that were relatively long lived (>20 weeks post-infection) [208], thus suggesting that cross-reactive antibodies are prevalent following infection. The molecular basis for this remains unclear but may be due to extensive sharing of polymorphic epitopes between PfEMP1 variants [159, 234]. In an early study, parasite isolates from ten Gambian children were tested in a checkerboard manner with the acute and convalescent sera collected from each child [226]. Most of the acute sera were not reactive towards the parasite isolate from that same child, whereas each of the serum samples collected during convalescence were highly reactive to the IE surface of the isolate from the same child but not from other children [226]. This study suggested that the VSAs expressed on the IE surface are highly diverse and children tend to acquire antibodies towards the variants expressed by the parasite causing that particular episode. Moreover, hyperimmune sera from Gambian adults agglutinated IEs from those ten children, suggesting that by adulthood most individuals have acquired a broad range of antibodies that protect against numerous parasite variants [226], and the presence of cross-reactive antibodies was also proposed. A large prospective study of young Kenyan children further showed that parasite variants expressed during episodes of clinical infection were less likely to be recognised by homologous sera collected before infection. This suggests that the variants are exploiting gaps in the pre-existing antibody repertoire in order to cause subsequent infections [227]. Consistent with that observation, antibodies from Sudanese children measured after the malaria season could agglutinate a broader range of isolates tested compared with antibodies measured before the season [235]. This suggests that natural *P. falciparum* infections are capable of inducing high antibody titres directed towards VSAs of the infecting parasite. In a cross-sectional study of Kenyan children during the low transmission season, antibodies to VSAs were higher in parasitaemic individuals, suggesting that these antibodies were induced by current infections [236]. Furthermore, plasma antibody levels were positively correlated with age, indicating an age-related and exposure-related component in the acquisition of VSA-specific antibodies [237].

The importance of antibodies to VSAs is evident through their role in mediating protective immunity to malaria (studies are summarised in Table 1). For example, in a longitudinal study conducted with young Gambian children, acquired antibodies to VSAs from several different isolates were consistently associated with protection from clinical malaria [228]. In Gabonese children, higher levels of IgG to VSAs were associated with lower rates of malaria [238]. A study of Ghanian children demonstrated that those with pre-existing antibodies before the malaria season were less likely to contract malaria than those with low levels of antibodies [239]. Furthermore, the presence of antibodies to a Ghanian isolate was significantly associated with protection from malaria [240]. However, the ability of sera from Kenyan children to recognise VSAs expressed by a Kenyan isolate was not associated with protection from malaria [227]. It appears that anti-VSA antibodies to some but not all parasite isolates are associated with protection [236, 239, 240], and presumably this depends on the prevalence of the parasite variant and its virulence properties. Nonetheless, evidence from all of the studies presented above supports the important contribution of anti-VSA antibodies to protection against malaria.

**Table 1:** Studies examining the association between human antibodies to VSAs and protection against malaria

Province, Country	Study	Population (*n*)	Age	Parasite isolates	Findings^a^
Farafenni, The Gambia	Marsh et al. [228]	Children (134)	<11 years	Gambian isolate (GAM83/1)	Antibodies to the IE surface were prospectively associated with protection against clinical episodes of malaria
Lambarene, Gabon	Tebo et al. [238]	Children (200)	6 months–11 years	IEs from 3 donor children	Convalescent sera from children with mild malaria had higher anti-VSA IgG compared to matched children with severe malaria
Dodowa, Ghana	Dodoo et al. [239]	Children (118)	3–15 years	IEs isolated from children	IgG to VSAs correlated with protection from clinical malaria
Daraweesh, Sudan	Giha et al. [240]	Adults and children (39)	5–50 years	IEs isolated from children	Antibodies to Ghanian isolate were significantly associated with protection
Kilifi, Kenya	Bull et al. [227]	Children (65)	1–5 years	Kilifi isolate (1759)	Lack of association between antibodies to the Kilifi isolate and protection from malaria
Kilifi, Kenya	Bull et al. [236]	Children (84)	1–5 years	IEs isolated from children	No association between anti-VSA antibodies or parasite positivity and protection from malaria
Lambarene, Gabon	Yone et al. [288]	Children (100)	1–8 years	IEs isolated from 6 donor children	Convalescent-phase IgG1 was associated with clinical protection
Kilifi, Kenya	Mackintosh et al. [289]	Children (272) Children (39)	6 months–10 years	Reference parasites (A4, 3D7), 1 clinical isolate from a Kenyan child	Failure to mount antibodies against these isolates was associated with malaria susceptibility in children with asymptomatic parasitaemia
Kilifi, Kenya	Chan et al. [102]	Children (296)	1–10 years	Reference parasites (3D7, E8B) and genetically-modified parasites	PfEMP1 is a dominant target of antibodies and PfEMP1-specific antibodies were associated with protection against symptomatic malaria

PubMed was searched for studies that examined the association between acquired human antibodies to total VSAs and protection against malaria, without an exclusion criterion, and attempts were made to include most studies

^a^Not all findings are listed for all studies

Despite the apparent role of PfEMP1 antibodies in mediating protection against malaria, the immense diversity of PfEMP1 limits its potential as a vaccine candidate. Although the repertoire of PfEMP1 variants is large, studies have suggested the expression of a dominant subset of variants that are restricted by their biological function in clinical disease. Immune sera from distinct geographic regions agglutinated IEs from other populations, suggesting that antibodies targeted cross-reactive epitopes expressed by many isolates [241]. Another study demonstrated that plasma antibodies were capable of recognising various parasite isolates regardless of the geographic origin of those IEs, suggesting that the repertoire of VSA-specific antibodies may be conserved over different populations [242]. A study in Kenya demonstrated that parasites isolated from children presenting with severe malaria were recognised by heterologous plasma antibodies, suggesting the expression of common PfEMP1 variants in this population [92]. Similarly, IEs from young Ghanian children with severe malaria were more commonly recognised by plasma antibodies from other children than those with uncomplicated malaria [243]. This suggests that a restricted subset of variants is expressed during severe disease to which antibodies are rapidly acquired [197]. These studies propose that the infecting parasites causing severe disease may be expressing a commonly expressed subset of VSA variants [236, 237]. A limitation of these studies is that they were only able to measure antibodies directed towards all VSAs expressed on the IE surface and not the proportion of antibodies to individual VSAs such as PfEMP1.

Little is known about antibody responses directed at antigens expressed on the surface of erythrocytes infected with gametocytes during their development in the human host. Such antigens could potentially elicit immune responses, similar to those of asexual parasites, which may result in the clearance of gametocytes in the host. Sera from Gambian children were reported to be highly reactive towards the surface of mature stage gametocyte-IEs but not towards immature stage gametocyte-IEs [244]. This may suggest that the antigens recognised by the serum antibodies in mature gametocyte-IEs are distinct from those expressed by immature gametocyte-IEs [244]. However, another study showed that sera from children in Papua New Guinea (PNG) were highly reactive to the surface of immature gametocyte-IE, similar to that observed with asexual trophozoite IEs, but not towards the surface of mature gametocyte-IEs [245]. These conflicting results demonstrate that further work is needed to better understand the antibody response directed towards antigens on the surface of gametocyte-IEs. Antibodies targeting these surface antigens represent potential vaccine candidates as they may mediate gametocyte clearance from the circulation, thus leading to reduced malaria transmission. More importantly, understanding the humoral response elicited by antigens on the surface of gametocyte-IEs will shed light on how these antibodies potentially act in synchrony with antibodies to other parasite stages to clear parasitaemia and reduce transmission.

### Human antibodies to PfEMP1

Epidemiological data have demonstrated that naturally acquired antibodies predominantly target variant-specific epitopes on the IE surface and PfEMP1 is thought to be a major antibody target. Lacking the molecular tools required to evaluate the significance of PfEMP1 independently of other VSAs, most studies have relied on the use of recombinant purified PfEMP1 domains to study human antibody responses to PfEMP1 (studies summarised in Table S2). A recent study in PNG used a DBLα protein microarray to demonstrate that the magnitude of the anti-PfEMP1 response was limited and variant specific in young children (<3 years of age), after which a broader spectrum of antibody recognition was achieved. By adulthood, serum antibodies were capable of recognising at least 20 different variants indicating an expansion of the PfEMP1 antibody repertoire [246]. Consistent with the preferential expression of PfEMP1 variants in severe disease [151, 152], the acquisition of anti-PfEMP1 antibodies by Tanzanian children was reported to be highly structured. Antibodies to different recombinant PfEMP1 domains were sequentially acquired, with children first acquiring antibodies to particular variants encoded by group A *var* genes [157, 247]. These findings were supported by another study whereby PfEMP1 DBLα domains were linked to young host age, disease severity and low levels of immunity [148]. Furthermore, they complement the finding that immunity to severe disease may be rapidly acquired after several infections [197].

Others have reported that while sera from Gabonese adults recognised most of the recombinant PfEMP1 based on the DBLα region, sera from children were less reactive to the different variants [248]. Interestingly, they also showed that antibodies from highly reactive adults chosen from previous assays were capable of recognising synthetic peptides based on conserved regions of DBLα [248], suggesting that antibodies to both variant-specific and conserved regions of PfEMP1 are co-acquired. Similarly, serum antibodies from Ghanian [239] and Sudanese [249] children recognised a recombinant peptide derived from a conserved epitope of the DBLα domain, with higher antibody levels observed in asymptomatic individuals compared to those with febrile malaria suggesting that antibodies against conserved epitopes of PfEMP1 may play a role in protective immunity. However, there was no association observed between these antibodies and protection from malaria in Ghanian children, because the recombinant peptide originated from a domain that is inaccessible to antibodies [239]. Since conserved epitopes are not consistent with being key antibody targets, the role of these antibodies in protective immunity remains unclear. Few studies have evaluated the protective effect of anti-PfEMP1 antibodies (studies summarised in Table 2) and results have been inconsistent. A longitudinal study with Ghanian children did not find a correlation between protection and antibodies to the DBLα domain of PfEMP1 [239]. No protective association was observed with antibodies to the recombinant PfEMP1 domains derived from the A4 parasite line [250].

**Table 2:** Studies examining the association between human antibodies to PfEMP1 and protection against malaria

Province, country	Study	Population (*n*)	Age	Antigen^a^	Findings^b^
Dodowa, Ghana	Dodoo et al. [239]	Children (118)	3–15 years	Recombinant DBLα domain	Plasma samples from most children recognised recombinant PfEMP1 No association between IgG to recombinant PfEMP1 and protection
Kilifi, Kenya	Mackintosh et al. [289]	Children	<10 years	Recombinant A4 PfEMP1 domains	Anti-DBLα antibodies in those who were parasite negative at baseline were associated with protection No association between antibodies to other domains and protection
Sudan	Staalsoe et al. [252]	Children	–	Synthetic peptides to conserved regions of PfEMP1 (same epitope as Dodoo et al. 2001)	IgG levels higher in asymptomatic infection compared to febrile malaria
Tanga, Tanzania	Magistrado et al. [290]	Children	0–19 years	Recombinant DBLα, DBL2γ, CIDR2β (3D7)	In children (4–9 years), the presence of antibodies were associated with reduced numbers of malaria episodes
Kilifi, Kenya	Chan et al. [102]	Children (296)	1–10 years	Reference parasites (3D7, E8B) and genetically-modified parasites	PfEMP1 is a dominant target of antibodies and PfEMP1-specific antibodies were associated with protection against symptomatic malaria

PubMed was searched for studies that examined the association between acquired human antibodies to recombinant PfEMP1 and protection against malaria, without an exclusion criterion, and attempts were made to include most studies

^a^Antibodies were measured by ELISA except for Chan et al. where antibodies were measured to native PfEMP1 by flow cytometry

^b^Not all findings are listed for all studies

**Fig. 2:**
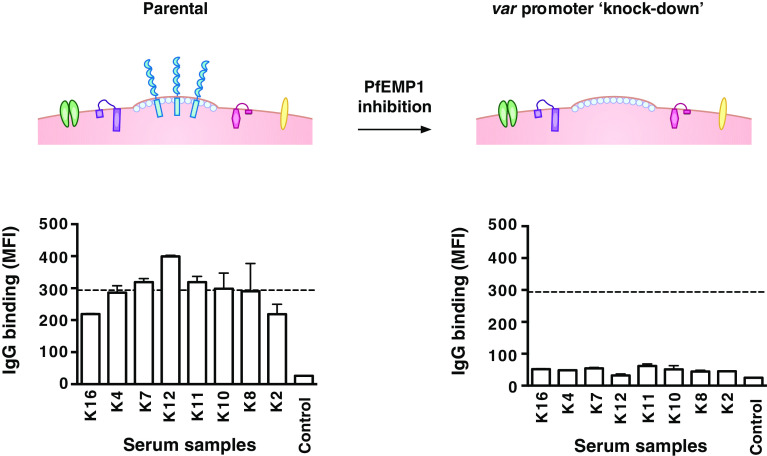
Evaluating the antibody response to PfEMP1 using transgenic *P. falciparum P. falciparum*-infected erythrocytes transfected with a construct that inhibits PfEMP1 expression but does not appear to have an impact on the expression of other VSAs (referred to as ‘*var* promoter knockdown’). This provides a novel approach to quantify antibodies to PfEMP1 and assess its importance as an immune target. The figure shows a representative selection of serum samples that were tested for IgG binding to parental and transgenic parasites [102]. Samples were from malaria-exposed Kenyan adults (K2-K16) and non-exposed Melbourne residents (Control). IgG binding to the surface of erythrocytes infected with the transgenic parasites was markedly reduced compared to parental parasites as previously reported [102]. The *horizontal dotted line* represents the mean level of IgG binding to parental parasites (*n* = 8); *bars* represent mean and range of samples tested in duplicate; IgG levels are expressed as geometric mean fluorescence intensity for both graphs

Quantifying the importance of different VSAs as targets of human antibodies is important for understanding immunity to malaria, but has been challenging to achieve. Recently, we developed a novel approach using genetically modified *P. falciparum* with inhibited PfEMP1 expression to evaluate the significance of PfEMP1 and other antigens as targets of acquired antibodies [102]. Suppressed PfEMP1 surface expression was achieved by the transfection of *P. falciparum* with a construct that encodes a *var* promoter without a downstream *var* gene [100, 101]. During in vitro culture with drug-selectable markers, the *var* promoter is expressed, which causes the silencing of all the other endogenous *var* promoters and thus suppresses PfEMP1 expression [100, 101] (Fig. 2). This approach was applied to human studies in Kenya to quantify serum antibodies to VSAs. We found that among malaria-exposed individuals, IgG binding to the surface of erythrocytes infected with the transgenic parasites was markedly reduced compared to that seen with parental parasites expressing PfEMP1. This suggests that the majority of the acquired human antibody response to the IE surface targets PfEMP1, while other VSAs appear to play a minor role as antibody targets. Our longitudinal studies further showed that individuals with PfEMP1-specific antibodies had a reduced risk of symptomatic disease while antibodies to other VSAs were not associated with protective immunity. Together, our findings demonstrate the significance of PfEMP1 as a major target of humoral immunity to malaria [102].

### Human antibodies to RIFIN and STEVOR

Data suggest that RIFIN and STEVOR may play significant roles as targets of malaria immunity; however, they have been little studied compared to PfEMP1. In an area of intense malaria transmission in Gabon, high levels of antibodies to recombinant RIFIN were detected in a majority of the adult population. Although RIFIN antibodies were also detected in children, the prevalence of these antibodies was much lower [179]. Despite the high copy number of *rif* genes, most adult sera were capable of recognising more than one RIFIN variant suggesting the generation of a large anti-RIFIN repertoire. In addition, elevated levels of RIFIN antibodies were associated with rapid parasite clearance in children [251]. Longitudinal studies with these children showed that although RIFIN antibodies were not correlated with a reduced rate of reinfection, RIFIN antibodies were long lived (~2 years) [251]. Furthermore, higher levels of RIFIN antibodies were detected in asymptomatic children than in those with severe disease, suggesting a protective effect of these antibodies [251]. The preadsorption of immune sera on recombinant RIFIN resulted in a marked reduction in the overall antibody reactivity to the IE surface [252]. This study proposed that in addition to PfEMP1, RIFIN is a key contributor to the overall anti-VSA response [252]. Others have shown that severe malaria patients in Ghana had substantially higher antibody levels to recombinant RIFIN than asymptomatic controls, suggesting the effect of antibody boosting during a malaria episode [253].

Little has been done on naturally acquired antibodies to STEVOR. Adult plasma had elevated levels of STEVOR antibodies suggesting the immunogenicity of STEVOR during a natural infection [254]. A longitudinal study with 9-month-old infants found no correlation between STEVOR antibodies and protective immunity, but revealed an increase in the frequency of parasitaemic episodes in those with high levels of antibodies [254]. The explanation for this observation remains unclear, but it is speculated that STEVOR is not involved in mediating immunity and acts as a marker of malaria exposure instead [254]. Further studies are necessary to elucidate the importance of antibodies against native STEVOR to fully understand its biological role. As noted above, our study using parasites with suppressed PfEMP1 expression demonstrated that PfEMP1 is the dominant target of human antibodies. However, a proportion of antibody reactivity to the transgenic parasites was observed, suggesting that antibodies to other VSAs, such as RIFIN and STEVOR, may still play an important role in immunity [102].

## Function of antibodies to VSAs

The mechanism by which antibodies to VSAs mediate protective immunity is only partially understood. Antibodies targeting VSAs are also thought to confer protection by interfering with IE sequestration or rosetting, features that contribute to malaria pathogenesis [79, 255]. Immune sera from infected *Aotus* monkeys blocked the binding of IEs to endothelial cells [18, 256]. Serum samples from pregnant women were capable of inhibiting IE adhesion to CSA [156, 257–259] and these antibodies were associated with improved birth outcomes in some studies [58, 260, 261]. In contrast, few studies have addressed adhesion inhibition in non-pregnant individuals. Convalescent serum from PNG children with symptomatic malaria inhibited the binding of homologous isolates to melanoma cells [230]. Antibodies from immune African adults inhibited the binding of a recombinant PfEMP1 domain to ICAM-1 [262]. Taken together, these results suggest the importance of antibodies that inhibit adhesion. Plasma antibodies from children presenting with mild malaria were capable of disrupting rosette formation in vitro, whereas those from children with severe malaria could not, suggesting that acquired antibodies are protective through rosette inhibition [79, 80, 150]. Furthermore, polyclonal antibodies against recombinant PfEMP1 domains disrupted existing rosettes and inhibited the formation of new rosettes [263].

Antibodies to VSAs also play a role in opsonising IEs for phagocytosis, an important mechanism of parasite clearance [264, 265]. A study with pregnant Malawian women showed that high levels of opsonising antibodies targeting VSAs correlated with parasite clearance and a decreased risk of maternal anaemia [266]. Immunisation of rabbits with recombinant PfEMP1 domains generated antibodies capable of inducing the opsonic phagocytosis of IEs [263]. Studies have also found that co-infection with HIV impaired the opsonic activity of antibodies for phagocytosis, thus leading to an increased risk of clinical malaria [267, 268]. Our recent data further identified PfEMP1 as a major target of naturally acquired antibodies that function to opsonise IEs for phagocytic clearance [102]. Individuals with high levels of antibodies to native PfEMP1 expressed on the IE surface promoted opsonic phagocytosis activity compared to transgenic parasites with inhibited PfEMP1 expression [102].

## Vaccine studies on PfEMP1

The importance of PfEMP1 as an immune target strongly supports the development of PfEMP1 as a major vaccine candidate. However, a major challenge to its development as a vaccine is substantial antigenic diversity. Studies with animal models have provided evidence that recombinant PfEMP1 is capable of mounting a protective immune response. Immunisation of *Aotus* monkeys with the CIDRα domain of PfEMP1 protected against a lethal parasite challenge with the homologous, but not the heterologous parasite strain [269]. To overcome the variant-specific limitations of PfEMP1 antibodies, studies have used different combinations of PfEMP1 domains to elicit a broader antibody response. Mice immunised with a combination of CIDRα domains developed antibodies capable of agglutinating IEs using various parasite lines [270, 271]. Using an in vivo model of *P. falciparum*-IE sequestration, rats immunised with diverse NTS-DBLα domains induced protective antibodies that reduced IE sequestration [272]. This was supported by a recent study in The Netherlands where naïve volunteers, who were infected with *P. falciparum*, generated cross-reactive antibodies that recognised PfEMP1 from different parasite genomes [273]. Taken together, these studies suggest that it may be possible to induce sufficient cross-reactive antibodies to protect against several PfEMP1 variants, provided that the specific combinations of domains are known. A recent study further demonstrated that rabbits immunised with different recombinant proteins based on the extracellular domains of PfEMP1 recognised native PfEMP1 on intact IEs [263]. These antibodies were also capable of inhibiting rosette formation and promoting the opsonic phagocytosis of IEs [263], suggesting that the inclusion of multiple domains is necessary for effective immunity.

Research efforts on PfEMP1-specific vaccines have centred on the DBLα domain because it is one of the most conserved domains of PfEMP1 and is involved in rosetting [274]. Immunisation of rats with recombinant protein based on the DBLα domain induced antibodies that recognised conserved PfEMP1 peptides [275]. However, these antibodies were not reactive towards the IE surface of intact, mature trophozoites or towards full-length PfEMP1 from different laboratory strains [275]. They were also unable to agglutinate different parasite lines or disrupt rosette formation [275]. Antibodies against recombinant DBLα were reported to inhibit rosette formation in another study [276]. The discrepancy between these two studies [275, 276] may be reflected by different methods of protein expression as the latter, but not the former, utilised protein refolding techniques to obtain conformational-dependent epitopes that may be necessary for antibody recognition [276]. Moreover, antibodies induced by a recombinant mini-PfEMP1 (DBLα-TM-ATS) disrupted preformed rosettes and prevented in vivo sequestration [277]. The importance of the DBLα domain was further supported by the marked reduction in IE sequestration in DBLα-immunised animal models [278].

The PfEMP1 variant, VAR2CSA, is a vaccine candidate for protection against malaria in pregnancy. High levels of antibodies to multiple VAR2CSA domains in acquired pregnant women through natural exposure were associated with reduced placental infection with *P. falciparum* [279, 280] in some studies. Furthermore, antibodies generated against full-length [160, 281] and single domains of VAR2CSA [282] by immunisation inhibited adhesion of IEs to CSA, suggesting that vaccine-induced antibodies may have a protective function in vivo.

**Fig. 3:**
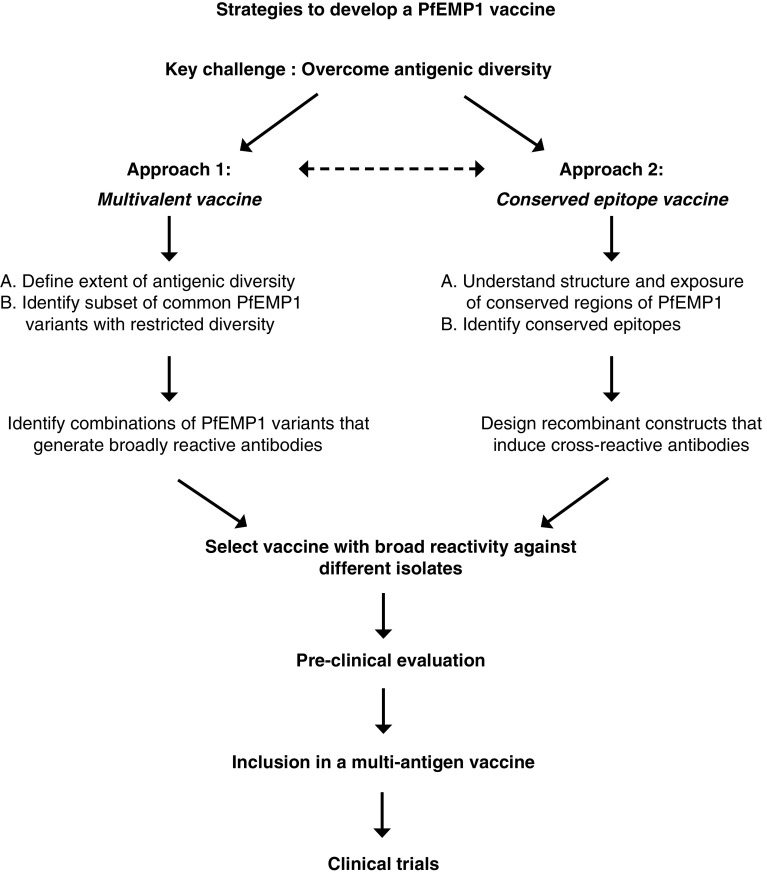
Approaches to overcome antigenic diversity of PfEMP1 in vaccine development. Antigenic diversity is the major challenge to developing PfEMP1 as a vaccine against malaria. The flow chart provides an overview of the two broad approaches to overcoming antigenic diversity in PfEMP1 and the steps involved in progressing vaccine candidates to the clinical trial stage. One approach is to develop a multivalent vaccine comprised of a mixture of common PfEMP1 variants that induces a broad repertoire of antibodies. A second approach is to identify conserved epitopes on PfEMP1 and develop a vaccine that targets these epitopes to induce broadly cross-reactive antibodies. As discussed in the text, there are significant challenges to overcome for each approach. It is likely that any PfEMP1 candidate vaccine antigen(s) would be included in a multi-antigen approach that includes antigens from other parasite life stages to ensure the development of a highly effective vaccine

A major obstacle in the development of PfEMP1 as a vaccine against *P. falciparum* malaria is its substantial level of antigenic diversity. Several strategies can be pursued as an approach to overcome antigenic diversity (reviewed in [283]; Fig. 3). One approach would be to develop a multivalent PfEMP1 vaccine that can induce a broad repertoire of antibodies against most variants. A priority of this approach would be to determine the extent of diversity in PfEMP1 and define a combination of PfEMP1 variants that is needed to generate a broad immune response (Table 3). This approach has been used successfully with the merozoite protein apical membrane antigen 1 to overcome antigenic diversity [284, 285]. Another approach would be to target conserved epitopes of PfEMP1 such that induced antibodies may recognise most PfEMP1 variants expressed. However, identifying conserved epitopes exposed on the IE surface and understanding the tertiary/quaternary structure of PfEMP1 remains highly challenging. Further studies and innovative approaches to target antibody responses towards conserved PfEMP1 epitopes are needed. Studies have demonstrated that naturally acquired cross-reactive antibodies do occur [232, 233] and can be induced by immunisation [271]. Additionally, defining effector mechanisms of PfEMP1 immunity and creating a reference panel of parasite isolates for the evaluation of vaccine candidates must be a priority. Of further importance is a detailed knowledge of the acquisition, boosting and maintenance of antibodies to PfEMP1, as this will impact on vaccine efficacy and durability, but only limited data are currently available. Ideally, malaria vaccines would induce long-lived protection via immune responses that were sustained for an extended period after vaccination and boosted after exposure. One study suggested that antibodies to some VSAs may be short-lived, whereas other responses are sustained [286]. A recent study in pregnant women suggested that antibodies to VAR2CSA may be maintained for several decades, whereas antibodies to merozoite antigens declined more quickly [287]. The durability of vaccine-induced immune responses is not well known, but is an important issue for the development of highly efficacious vaccines against malaria (Table 3).

**Table 3:** Research priorities for the development of PfEMP1 vaccines

General priorities	Define effector mechanisms of PfEMP1 immune responses and quantify their importance Understand how antibodies to PfEMP1 are acquired, boosted and maintained over time Define antigenically conserved and diverse regions of PfEMP1 Create a reference panel of isolates for testing/evaluating vaccine candidates
Development of a multivalent vaccine	Determine the extent of local/global antigenic diversity in PfEMP1 Understand the evolution of diversity that may lead to vaccine escape Define the number of variants/domains to be included Identify specific domains of PfEMP1 for possible vaccine inclusion
Development of a vaccine targeting conserved epitopes	Understand the tertiary and quaternary structure of PfEMP1 Identify conserved epitopes exposed on the surface of IEs Create innovative approaches/technologies for identifying and targeting conserved epitopes

It is likely that a highly effective malaria vaccine will require a multi-antigen, multi-stage approach. Therefore, it is anticipated that any PfEMP1-based vaccine antigens would have to be included as part of a vaccine containing antigens from other stages of the parasite life cycle, such as merozoite antigens to enhance blood-stage immunity and circumsporozoite protein for induction of pre-erythrocytic immunity, and the inclusion of gametocyte antigens for transmission-blocking immunity. We suggest that after lead PfEMP1-based vaccine antigens have been identified and prioritised, they will then need to be evaluated in combination with other vaccine antigens before proceeding further to clinical trials.

## Conclusion

Understanding the targets and mechanisms of human immunity is crucial for informing and advancing the development of highly effective malaria vaccines and for developing tools for measuring immunity and exposure in populations to help evaluate the impact of malaria control interventions and identify populations at risk of malaria. Multiple studies in different populations now provide strong evidence that IE surface antigens, or VSAs, are important targets of acquired protective immunity. The most important of these antigens is PfEMP1, which is a major virulence factor enabling vascular adhesion and sequestration of IEs. Studies are beginning to identify specific variants of PfEMP1 that may be common in populations or linked to disease pathogenesis and that may be targeted in vaccine development. However, there are still major gaps in our knowledge on this topic, and these are important questions for future research. Little is known about other known or proposed surface antigens and their significance as targets of immunity and new strategies, and approaches are needed to clearly define their significance in immunity. Similarly, knowledge on surface antigens of IEs with *P. vivax*, the second major cause of malaria, is very limited. The role of surface antigens on gametocyte-IEs needs to be determined, as antibodies to these antigens may help clear gametocyte-IEs and thereby reduce malaria transmission; currently there is great interest globally in transmission-blocking vaccines, but there are few strong candidates in development. A greater understanding of effector mechanisms that mediate immunity is needed, including both humoral and cell-mediated responses, and additional assays to measure antibody functional activity in studies of acquired immunity and in vaccine trials would be valuable. Finally, strategies to overcome antigenic diversity in PfEMP1 would provide an exciting new opportunity in malaria vaccine development.

### Electronic supplementary material

Below is the link to the electronic supplementary material.

Supplementary material 1 (DOCX 32 kb)

## Abbreviations

ATS: Acidic terminal segment of PfEMP1
CD36: Cluster of differentiation 36 receptor
CIDR: Cysteine-rich interdomain regions
CMI: Cell-mediated immunity
CR1: Complement receptor 1
CSA: Chondroitin sulphate A
DBL: Duffy binding-like domain
HA: Hyaluronic acid
ICAM-1: Intercellular adhesion molecule 1
IE: *P. falciparum* infected erythrocyte
KAHRP: Knob-associated histidine-rich protein
MAHRP1: Membrane-associated histidine-rich protein 1
MC: Maurer’s clefts
MESA: Mature infected erythrocyte surface antigen
PfEMP1: *P. falciparum* erythrocyte membrane protein 1
PfMC-2TM: *P. falciparum* Maurer’s clefts two transmembrane protein
REX: Ring exported proteins
RIFIN: Repetitive interspersed family proteins
STEVOR: Subtelomeric variable open reading frame proteins
SURFIN: Surface-associated interspersed gene family proteins
VSA: Variant surface antigen
