# Supplementary material for: Surface antigens of Plasmodium falciparum-infected erythrocytes as immune targets and malaria vaccine candidates
Source: Cell Mol Life Sci. 2014 Apr 2;71(19):3633–57. doi: 10.1007/s00018-014-1614-3 (PMC4160571; doi:10.1007/s00018-014-1614-3)
Supplement: Supplementary file 1 — Supplementary material 1 (DOCX 32 kb) [file 18_2014_1614_MOESM1_ESM.docx]

**Supplemental Materials:**

Surface antigens of *Plasmodium falciparum*-infected erythrocytes as immune targets and malaria vaccine candidates

Jo-Anne Chan, Freya J.I. Fowkes, James G. Beeson

Cellular and Molecular Life Sciences, 2014

**Table S1 Studies examining naturally acquired human antibodies to VSAs expressed on the IE surface**

| Province, Country | Study reference | Population (n) | Assay^1^ | Parasite isolates | Findings^2^ |
| --- | --- | --- | --- | --- | --- |
| Punjab, Pakistan | Iqbal et al 1993  [229] | Children (15) | Agglutination | IEs isolated from children | Children developed isolate-specific antibodies during convalescence |
| Madang, Papua New Guinea | Forsyth et al 1989 [230] | Children (21) | Agglutination | IEs isolated from children | All isolates were recognized only by homologous convalescent sera |
| Farafenni, The Gambia | Newbold et al 1992 [231] | Adults (5; pooled sera) | Mixed agglutination | IEs isolated from children and lab isolates | Antibodies are predominantly variant-specific and cross-reactive antibodies are rare |
| Fajara, The Gambia | Marsh and Howard 1986 [226] | Children (10) | Agglutination | IEs isolated from children | Convalescent sera reacted with homologous, but not heterologous parasite isolates |
| Sundargarh, India | Chattopadhyay et al 2003 [232] | Adults | Mixed agglutination | IEs isolated from donor patients | Confirmed the presence of both variant-specific and cross-reactive antibodies in convalescent sera |
| Brisbane, Australia | Elliott et al 2007 [208] | Adult returned travellers with single *Pf* infection | Flow cytometry | Lab (ItG, 3D7, HM) and clinical isolates (Pf2004, Pf2006) | Acute infections may be sufficient to induce cross-reactive antibodies towards heterologous parasite isolates |
| Kilifi, Kenya | Bull et al 1998 [227] | Children (65) | Agglutination | IEs isolated from children | Parasite variants expressed during clinical disease corresponds to the gap in pre-existing antibody repertoire |
| Daraweesh, Sudan | Giha et al 1998 [235] | Children (64) | Agglutination | IEs isolated from 5 donor children | Post-season samples agglutinated more isolates than pre-season or acute samples |
| Kilifi, Kenya | Bull et al 2002 [236] | Children (1106) | Agglutination | IEs isolated from children in previous study (Bull et al., 1998) | Proportion of children with anti-VSA antibodies were higher among those with concurrent infection |
| Dodowa, Ghana | Ofori et al 2002 [237] | Children (150) | Flow cytometry | IEs isolated from 12 children | Antibody acquisition increases with age and exposure |
| Africa | Aguiar et al 1992 [241] | Adult sera from African countries, Asia & South America | Agglutination with IEs from East & West Africa | Clinical isolates from African children | Sera from immune adults agglutinated IEs from different geographical regions |
| Indonesia Ghana Tanzania Sudan | Nielsen et al 2004 [242] | Adults (40) Children (96)  Children (45)  Adults and children (57) | Flow cytometry | IEs isolated from children | Antibodies reacted with isolates from different geographical region |
| Kilifi, Kenya | Bull et al 1999 [92] | Children (33) | Agglutination | IEs isolated from children | Parasite isolates highly recognized by heterologous plasma |
| Accra, Ghana | Nielsen et al 2002 [243] | Children (100) | Flow cytometry | IEs isolated from children | IEs from children with severe malaria more commonly recognized by plasma antibodies than IEs from uncomplicated disease |
| Kilifi, Kenya | Bull et al 2000 [291] | Children (115) | Agglutination | IEs isolated from children | IEs from severe malaria patients highly recognized by heterologous plasma compared to IEs from mild malaria patients |
| Kilifi, Kenya | Kinyanjui et al 2003 [286] | Children (46) | Agglutination and flow cytometry | IEs isolated from children | IgG3 to VSAs were highest compared to IgG1, IgG2 and IgG4  Early responses predominantly IgM |
| Lambarene, Gabon | Cabrera et al 2004 [292] | Children (100), adults (21) | Flow cytometry | IEs isolated from 6 donor children | Semi-immune adults :  IgG3>IgG2>IgG1=IgG4  Children : IgG3 similar to IgG4 and higher than both IgG1 and IgG2 |
| Lambarene, Gabon | Yone et al 2005 [288] | Children (200) | Flow cytometry | IEs isolated from 6 donor children | Acute infection mostly IgG1 and IgG3 |
| Daraweesh, Sudan | Giha et al 1999 [293] | Adults and children (29) | Agglutination and flow cytometry | IEs isolated from 4 donors | Antibodies recognized VSAs expressed by parasites of different genotypes |
| Madang, Papua New Guinea | Reeder et al 1994 [294] | Children (32) | Agglutination and adhesion | IEs isolated from children | Isolates were generally recognized only by homologous convalescent sera and all isolates formed rosettes |
| Blantyre, Malawi | Beeson et al 1999 [56] | Children and pregnant women | Agglutination and adhesion | IEs from children and pregnant women | Isolates from children and pregnant women have distinct antigenic and adhesive properties |

Note: PubMed was searched for studies that measured acquired human antibodies to total VSAs without an exclusion criteria and attempts were made to include most studies.

^1^Antibody binding measured to whole IEs using agglutination assays or flow cytometry

^2^Not all findings are listed for all studies

**Table S2 Studies examining naturally acquired human antibodies to PfEMP1**

| Province, Country | Study | Population (n) | PfEMP1 domains | Assay | Findings^1^ |
| --- | --- | --- | --- | --- | --- |
| Madang, Papua New Guinea | Barry et al 2011 [246] | Children (40) and adults (40) | Partial DBLα domain expressed in *E. coli* | Protein microarray | Antibody response in children is limited and variant specific, and levels increase with age. |
| Muheza, Tanzania | Cham et al 2010 [247] | Children (672) | DBL domains (32 constructs) expressed with baculovirus in insect cells | Luminex microsphere system | Antibody responses are highly structured and antibodies to a given domain are acquired before antibodies to other domains |
| Muheza, Tanzania | Cham et al 2009 [157] | Children (1342) | DBL domains (48 constructs) expressed with baculovirus in insect cells | Luminex microsphere system | Children first acquire antibodies to PfEMP1 domains encoded by group A *var* genes |
| Lambarene, Gabon | Oguariri et al 2001 [248] | Semi- or non-immune individuals | DBLα domain expressed in *E. coli* | ELISA | Antibody response predominantly directed at variable epitopes of DBLα |
| Kilifi, Kenya | Moll et al 2007 [278] | Children (58) | DBLα domain expressed in *E. coli* | Flow cytometry | High level of antibody recognition by semi-immune children sera |
| Kilifi, Kenya | Mackintosh et al 2008 [289] | Adults and children | A4 PfEMP1 domains expressed in *E. coli* | ELISA and flow cytometry | Greater recognition of DBLα and DBLγ compared to other domains  Children who were parasite positive were more likely to recognize more domains |
| Sundargarh, India  Manhica, Mozambique | Mayor et al 2009 [276] | Adults and children (644)  Adults (105) and children (60) | DBLα domain expressed in *E. coli* | ELISA | IgG response was low in areas of low endemicity but high in intense endemicity, and antibody acquisition is rapid and age-related |
| The Netherlands | Turner et al 2011 [273] | Naïve volunteers infected with *Pf* NF54 | DBL and CIDR domains (104 different constructs) expressed with baculovirus in insect cells | Luminex microsphere system | Short course of infection led to acquisition of cross-reactive antibodies to numerous PfEMP1 variants |
| Kambila, Mali | Crompton et al 2010 [295] | Adults and children (225) | - | Protein microarray | Children who did not contract malaria during the season had higher levels of antibodies to *Pf* proteins (including PfEMP1) |

Note: PubMed was searched for studies that measured acquired human antibodies to PfEMP1 without an exclusion criteria and attempts were made to include most studies.

^1^Not all findings are listed for all studies
